# Interpretive Flexibility in Mobile Health: Lessons From a Government-Sponsored Home Care Program

**DOI:** 10.2196/jmir.2816

**Published:** 2013-10-30

**Authors:** Jeppe Agger Nielsen, Lars Mathiassen

**Affiliations:** ^1^Aalborg UniversityDepartment of Political Science, Center for Organization, Management & AdministrationAalborgDenmark; ^2^J Mack Robinson College of BusinessCenter for Process InnovationGeorgia State UniversityAtlanta, GAUnited States

**Keywords:** home health care, mobile health, mobile technology, implementation process, government sponsorship, case study

## Abstract

**Background:**

Mobile technologies have emerged as important tools that health care personnel can use to gain easy access to client data anywhere. This is particularly useful for nurses and care workers in home health care as they provide services to clients in many different settings. Although a growing body of evidence supports the use of mobile technologies, the diverse implications of mobile health have yet to be fully documented.

**Objective:**

Our objective was to examine a large-scale government-sponsored mobile health implementation program in the Danish home care sector and to understand how the technology was used differently across home care agencies.

**Methods:**

We chose to perform a longitudinal case study with embedded units of analysis. We included multiple data sources, such as written materials, a survey to managers across all 98 Danish municipalities, and semistructured interviews with managers, care workers, and nurses in three selected home care agencies. We used process models of change to help analyze the overall implementation process from a longitudinal perspective and to identify antecedent conditions, key events, and practical outcomes.

**Results:**

Strong collaboration between major stakeholders in the Danish home care sector (government bodies, vendors, consultants, interest organizations, and managers) helped initiate and energize the change process, and government funding supported quick and widespread technology adoption. However, although supported by the same government-sponsored program, mobile technology proved to have considerable interpretive flexibility with variation in perceived nature of technology, technology strategy, and technology use between agencies. What was first seen as a very promising innovation across the Danish home care sector subsequently became the topic of debate as technology use arrangements ran counter to existing norms and values in individual agencies.

**Conclusions:**

Government-sponsored programs can have both positive and negative results, and managers need to be aware of this and the interpretive flexibility of mobile technology. Mobile technology implementation is a complex process that is best studied by combining organization-level analysis with features of the wider sociopolitical and interorganizational environment.

## Introduction

Health care information technology (HIT) has the potential to produce increased quality and efficiency of service delivery [1]. However, HIT implementation is not a straightforward process. It is often as messy as it is exciting, and at times, it may turn into a battlefield where progress occurs through a combination of both “muddling through” and rational decision making [2,3]. Accordingly, when managers implement mobile health, they will likely face both opportunities and challenges.

Mobile health, or mHealth, involves “emerging mobile communications and network technologies for healthcare systems” [4]. The hardware includes laptops, personal digital assistants (PDAs), and smartphones, with more advanced devices integrating and combining functionality [5]. Mobile devices are increasingly involved in many aspects of health care delivery [5-8] because they offer great benefits compared to using personal computers—most importantly the capacity to access information and complete various functions in real time at the point of care [9-11]. Despite these obvious advantages, studies have raised issues related to implementing mobile health care [12-19].

Some researchers have found that the uptake of mobile health systems is more limited than what one might expect from the optimistic tone in the field [12]. Others have highlighted privacy concerns [13], end-user resistance to change [14], lack of adequate training and management support [15], and technical issues suggesting more attention should be given to the overall architecture of the mobile health system and to user interfaces [16]. Another study illustrated how users found laptop computers easier, faster, and more satisfying to use than handheld computers in the data recording process [17]. It has also been demonstrated that mobile devices provide a reservoir of bacteria known to cause infections within the hospital area [8]. Although these studies have enriched our understanding of the impact of mobile health systems, they most often report from pilot projects or from very restricted contexts.

Against this backdrop, the objective of this study was to contribute to mHealth research by examining a large-scale mHealth implementation project in the Danish home care sector. In this context, mobile technology has spread quickly since the mid-2000s, and today most home care agencies have invested in PDAs or smartphones for their health care personnel. Drawing on multiple sources of data covering the period 1998-2008, we demonstrate how mobile technology implementation offered new opportunities and challenges as key stakeholders debated visions for use of mobile technology within the Danish home care sector and transformed health care practices in individual home care agencies (HCAs).

Mobile health dates to the 1990s. The PDA was introduced by Apple in 1993 and became a household product by the end of the 1990s [20]. Parallel to this progress, mobile devices started being used in health care settings in a number of western countries [11]. For instance, in Danish home care, pilots were initiated in 1998 as some agencies tested mobile devices among care workers and nurses. Today, PDAs and smartphones are widely used by health care professionals in most OECD countries [20], and expectations of the transformative potential of mHealth are massive [21] as mobile technologies represent promising new ways in assisting health care professionals as they access, manage, and share critical information at the point of care.

The fact that mobile devices are used by an increasing number of primary staff in health care has attracted considerable research attention. Early studies of mHealth focused on its potential benefits, opportunities, and barriers [22,23]. Comprehensive literature reviews found that mobile devices were widely used by health care professionals and that their use was expected to increase significantly in the years to come [6,20]. Rothschild et al examined US doctors’ use of mobile technology, concluding that doctors found them “to improve patient care and be valuable in learning of recent alerts and warnings” (p. 619 [10]). Overall, mHealth has been shown to improve communication among health care staff [5].

More recently, investigations have presented a set of factors that help explain the adoption of mobile health care systems [7,24-28]. Park and Chen emphasized perceived usefulness and perceived ease of use as key factors for both physicians and nurses in their use of smartphone technology [24]. Similarly, Zhang et al concluded that nurses’ view of usefulness is the main factor in the adoption of mobile technology [25]. Some Scandinavian studies have focused on mobile technology in home care [29]. In a study of Finnish home care agencies, Vuokko illustrated how the introduction of mobile technology impacts home care work and creates concerns among staff related to issues of control, surveillance, and distrust of the management while at the same time, they see benefits in terms of better coordination and documentation [29]. Finally, researchers have started to investigate factors that impact mHealth adoption, usage, and channel preferences from a client perspective [21]. In this line of research, it is demonstrated how mHealth applications can empower clients to track and manage their own health [30].

We go beyond this literature by trying to understand the processes through which mHealth is adopted and implemented into health delivery practices [31-33]. Process-oriented approaches can reveal important lessons on how to manage new technologies, and they have previously been used with success to address the complexities involved in HIT implementation [1,34,35]. These studies suggest technologies have interpretive flexibility [36] as various stakeholders construct the meaning of the technology differently. We assume such an approach may prove useful in exploring mHealth by emphasizing differences in how stakeholders perceive the nature of technology, technology strategy, and technology use [37].

Conceptualizing process “as a sequence of events that describes how things change over time” [33], we emphasize that change may very well unfold differently depending on the context in which organizations are embedded. Following Newman and Robey [38] and Langley [39], we distinguish between antecedent conditions, key events, and practical outcomes and use temporal bracketing to highlight the important phases through which the process unfolds. Process data, in particular from longitudinal studies, are indeed complex. It is therefore important to adopt analytical approaches that can help manage this complexity and bring forward valuable insights and lessons. Contextual considerations are also highly relevant in process studies [32,40] as they can help us understand how organizational implementation of HIT, such as mHealth, is shaped by the wider sociopolitical and interorganizational environment [41-43]. This adds further to the number of variables studied, suggesting a case study design with in-depth examination of how the context and the contents of HIT implementation are formed over time [44,45].

## Methods

The research is organized as a longitudinal case study with embedded units of analysis of the implementation of mobile technology into Danish home care agencies. We examined how mobile technology implementation unfolded in three specific home care agencies and complemented this organization-level analysis with a perspective of the broader home care sector by including an examination of how government, IT vendors, and interest groups were involved in shaping the implementation process. We followed the implementation process over a 10-year period from 1998 when the first initiatives were taken to 2008 when technologies were in use in the majority of Danish municipalities.

The Danish health care sector is organized into 5 regions with responsibility for hospitals and 98 municipalities which, according to the Danish Social Services Act, are responsible for home care to help the elderly and disabled cope with everyday life [46]. Even though clients can choose private providers and have the costs compensated by government (the so-called Free Choice model), home care services are predominately delivered by the public sector [47]. Home care services are long-term or temporary. Long-term home care is provided free of charge while citizens may be asked to subsidize the costs of temporary home care depending on level of income [48].

There are approximately 700,000 people over the age of 67 in Denmark. Of these, more than 160,000 (2011) receive long-term home care and a further 8000 people receive temporary home care. Home care involves daily-living assistance in clients’ own homes including a wide range of nursing and care services such as coordination with post-acute care, assistance with medication, personal hygiene and care, cleaning, shopping, and preparation of meals. In Denmark, the average duration of hospitalization has been remarkably reduced, which means that the home care sector has experienced increasingly complex tasks. Internationally, Denmark is rated as a leader in the area of home care services with the most far-reaching public-financed services for the elderly. Approximately 70,000 full-time care workers and 6000 nurses are employed in the sector [49].

We collected primary data between June 2007 and May 2008, beginning with fly-on-the-wall observations as the first author followed nurses and care workers “at work with PDAs” in a specific home care agency. Although the lessons learned from this pilot are not directly part of the data sources in this article, these initial observations provided valuable insights and improved our understanding of the research context and key stakeholders involved. For this study, we used multiple data sources, including written materials, a survey to managers across all 98 Danish municipalities, combined with semistructured interviews with managers, care workers, and nurses in three selected home care agencies, as summarized in Table 1. (See Multimedia Appendix 1 for more information about the three cases.)

**Figure 1:**
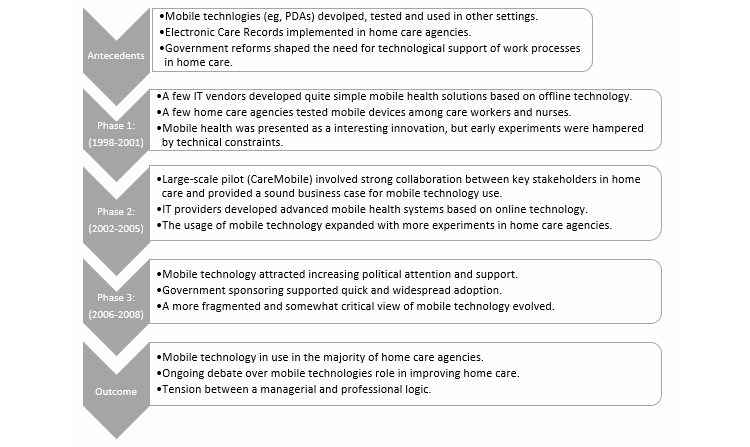
Implementation of mobile health in the Danish home care sector.

Interviews were tape-recorded and transcribed. Using all our data, we constructed a chronology of the major events that took place during 1998-2008 [39,50]. Also, antecedent conditions and outcomes were identified [38,40] as summarized in Figure 1. To improve reliability, the analysis was presented to key informants in the home care sector and consequently revised [51].

Below, we present the results of our analyses in two steps. First, we provide an overview of the implementation process in the home care sector as summarized in Figure 1. Next, we provide detailed insights into the implementation of mobile technology in three selected home care agencies (municipalities).

**Table 1:** Data sources.

Data sources	Description
Survey to home care managers in all 98 Danish municipalities to track the adoption rate of mobile technology	Managers were interviewed over the phone in June 2007, which enabled a 100% response rate. We used a structured interview guide and inquired about: How many home care agencies are using mobile technology? When did they start using the technology? Which groups of employees make use of mobile technology? We also inquired about their motives for adopting or rejecting the technology, sources of inspiration, and the importance of governmental subsidies.
Written materials and interviews with key stakeholders in the home care sector	We reviewed government, consultants, and vendor websites for available written materials as a way to further our understanding of how mobile technology implementation was shaped in interplay with the broader context.
	We interviewed representatives from Local Government Denmark (LGDK, a major interest group for municipalities), the Ministry of Social Affairs, and Ministry of Finance as they were leading in the discussion on innovating home care by using mobile technology. The interviews were conducted in May 2008 and lasted on average about 1 hour.
Three cases of mobile technology implementation within specific home care agencies (HCA 1, HCA 2, & HCA 3).	The three selected home care agencies had each used mobile technology for some time (respectively 5, 2, and 2 years), which allowed us to achieve comprehensive insight into the implementation process and how the technology was used in day-to-day practices.
	We conducted semistructured interviews in each case with key stakeholders: managers and employees using mobile technology (nurses and care workers). In total, 10 managers or project managers (respectively 4, 4, and 2 in each case) and 24 employees (respectively 7, 8, and 9 in each case) were interviewed. We organized a protocol to structure the interview process and personalized it for specific stakeholder groups. For instance, the protocol for managers included questions that permitted the managers to express how they perceived the nature of mobile technology, the implementation strategy, and mobile technology in use, but also who they saw as the major sources of inspiration and their collaboration with IT vendors and other stakeholders.
	We conducted a survey of care workers across the three selected home care agencies (N=315, response rate 63%), particularly focusing on care workers’ perceptions towards the mobile technology and how they use the technology in daily practice.
	Written documents (eg, project descriptions, minutes from meetings, and evaluations) were collected in each case. While interviews enlighten the more informal processes and struggles surrounding mobile technology implementation, written documents identified the formal motives behind mobile technology implementation.

## Results

### Process Analysis

#### Antecedents

Danish home care has been through a series of New Public Management-inspired [52] reforms since the mid-1990s aiming to improve accountability and efficiency [47]. These reforms shaped the need for technological support of work in home care, for example, to meet requirements for transparency and more accurate documentation. The first important review of IT usage in home care was carried out in 1994. The study found that IT systems were used on a very small scale, and roughly 90% of all administrative tasks were handled manually [53].Yet, the possibility of using new technology in home care was boosted in the mid-1990s as the Common Language reform established standards and data models, which became common to all IT vendors providing electronic care records and mHealth systems in home care agencies [54]. Accordingly, home care agencies increased their IT usage during the 1990s; for instance, the majority implemented electronic care records. These systems are tied to the electronic medical record systems in hospitals and include a comprehensive database with client information. Based on these databases, mHealth systems were developed giving health care personal access to client information at the point of care. Since the first tests in the late 1990s, more sophisticated devices were implemented as the mobile network increasingly involved online solutions as a replacement for offline solutions. Whereas offline solutions imply that care workers download and upload client data to mobile devices at the office, online solutions afford access to and the update of centrally stored client data in real-time at the point of care.

#### Phase 1 (1998-2001)

Initial experiments with mobile technology occurred in the late 1990s and have expanded significantly since. The first home care agency (Municipality of Græsted-Gilleleje) started in 1998 as a group of nurses and care workers tested handheld devices. IT vendors and consultants played a decisive role in this early phase. They developed technological solutions based on offline connectivity and worked closely with early adopter organizations by actively engaging in the implementation of pilots. Mobile technology was promoted by IT vendors and managers as an interesting innovation and a fresh way to modernize public home care, but despite optimistic announcements, projects were hampered by technical difficulties, and initial projects were suspended after pilots.

#### Phase 2 (2002-2005)

Whereas IT vendors and consultants inspired home care agencies early on in the change process, government bodies started to play a more vital role in the next phase. IT vendors started to develop more advanced mHealth systems based on online technology, but it was a large-scale sector-wide pilot, CareMobile, that was launched in 2002 and reported in 2005 that positioned mHealth on the wider political agenda [55]. The CareMobile project was managed in collaboration between the Ministry of Finance, Ministry of Social Affairs, and LGDK, plus it included several IT vendors and consultancy companies as well as six pilot municipalities. CareMobile offered a sound business case for mobile technology adoption, and the final evaluation highlighted that the technology was mature and that investment could be gained in 1 year, as meeting activities and duplicate data entry could be avoided [55]. It was estimated that administrative tasks in home care could be reduced by more than 3000 full-time positions if mobile technology were adopted by all municipalities [56,57]. Accordingly, the usage of mobile technology expanded in this phase with more experiments in home care agencies. mHealth was put on the political agenda, most prominently through the CareMobile project. Furthermore, CareMobile served as an important activity in building legitimacy for mobile technology adoption and use.

#### Phase 3 (2006-2007)

While mobile technology until 2005 was reserved for an exclusive group of home care agencies, the following years resulted in widespread adoption. In 2007, 76 of 98 municipalities (78%) had adopted mobile technology and another 13 (13%) expected to implement in 2008 (see Multimedia Appendix 1). In this phase, mHealth gradually attracted more political consideration and support. In the ICT strategy for the social sector, mHealth was presented as a high priority area [58]. Key ministers, including the Prime Minister, emphasized on several occasions the benefits of mobile technology [59]. Finally in 2006, the adoption of mobile technology started to accelerate when the government decided to support implementation with approximately €45 million. The positive results from the CareMobile project provided the rationale for allocating government subsidies to mHealth [60]. Subsequently, 66% of all municipalities responded that government funding had decisive importance for their adoption of mobile technology (Table 2). In this sense, the sponsoring activity was very effective. Whereas the earlier phases were characterized by a high degree of consensus (“mobile health is good”), this phase provided a more fragmentary view of mHealth. Consistent with the rapid dissemination of the technology, the powerful DaneAge Association and the trade unions articulated rather critical views emphasizing that mHealth was associated with unnecessary organizational control [61].

**Table 2:** The importance of government funding for mobile technology adoption (%) (source: Survey to home care managers in all 98 municipalities).

	Yes	No	No answer	Sum	N
Has government funding been of decisive importance for mobile technology adoption?	66	30	4	100	76^a^

^a^The number of municipalities that had adopted mobile technology at the time of the survey (2007).

#### Outcome

In 2008 when our study ended, the vast majority of home care agencies had adopted mobile technology, and ministries, consultants, and IT vendors still supported the use of mobile technology. Yet, the many and varied experiences of transforming the new technology into new practices continued to influence the ongoing debate over mHealth’s role in improving Danish home care. More critical reports appeared describing control and monitoring issues in the practical use of mobile technology and illustrating a more general tension between management and professionalism in home care. While the introduction of the technology at the outset appeared remarkably promising, many projects were hampered by technical difficulties that also likely contributed to an increased resistance from health care personal.

### Case Analyses

#### Summary

To provide detailed insights into the changes that resulted in individual home care agencies, we trace the implementation of mobile technology in three case settings. These cases highlight the interpretive flexibility [37] of mobile technology as summarized in Table 3. Multimedia Appendix 1 includes data tables about mobile technology implementation and use in these three settings.

#### Home Care Agency 1

This agency implemented mobile technology (Nokia Communicator was the chosen hardware) in 2005 based on online technology. The new system was implemented with Zealand Care as the vendor and responsible for training sessions. Government funding was not of decisive importance for initial investment by HCA 1 in mobile technology, but the CareMobile initiative served as a major source of inspiration. Mobile technology was considered a useful coordination platform for sharing information, for reducing administrative tasks, and especially for decreasing meetings in home care. A manager explained their technology strategy:

We could see an advantage as each nurse and care worker had a cell phone at the point-of-care, and we would like to cut down on the time we spent at meetings. In fact, it was a demand from our politicians in the City Council that if we adopted more technology, we had to cut down our meeting activity.

Working practices changed substantially as strategies for mobile technology were implemented and transformed into daily work (technology in use). Mobile devices were used for documentation (especially registration of time and services provided), for internal communication between managers, nurses, and care workers and to access information at the point of care. The morning joint meetings at central offices were eliminated as health care personnel instead based their working day on information exchange through mobile communication. However, not all face-to-face meetings were canceled, and joint coordination meetings were held in the afternoon twice a week. Contrary to the initial purpose, mobile technology was not used for filling out records. Instead, the health care personal preferred desktop computers with larger screens and larger keyboards for this specific activity.

Care workers received mobile technology with skepticism, particularly due to the canceling of the morning meeting. In contrast, managers viewed mobile technology in positive terms and had a clear vision of what benefits mobile technology could bring to the organization. Although the care workers’ perceptions became more positive over time, mixed attitudes towards mobile technology were still apparent after 2 years of use. It was not the technology itself that created debate (it was perceived as easy to use) but the reduction in meeting activity that proved most controversial as it was considered a reduction in knowledge sharing and collegial relationships. One care worker expressed: “I miss the morning session. I do miss the social and collegial contacts.” Overall, mobile technology implementation in HCA 1 demonstrates how the new technology impacted day-to-day working routines (eg, communication and access to information) and conflicted with perceived advantages of established practices (eg, morning meetings).

#### Home Care Agency 2

This agency also implemented an mHealth system with online connectivity in 2005. The system was provided by Ramböll, and PDAs were the selected hardware including telephone features. Government funding was not of decisive importance for the decision by HCA 2 but sponsoring did help roll out the technology throughout the organization. HCA 2 took a different approach to mHealth compared to HCA 1. The technology strategy was not so much to support a cost-saving agenda, but more to promote a modern image of home care. A home care manager stated: “It was very much related to status...to give our staff advanced mobile technology will certainly raise the status...In many home care agencies it’s about efficiency, saving and control...this has not been the case here.”

HCA 2 did not mandate detailed time registration and abolish joint morning meeting (as in HCA 1). Instead, it was up to each home care unit whether they wanted to use the technology for these purposes. This more lenient implementation strategy appeared to influence the care workers’ interpretation of municipal control as being weak compared to HCA 1 and HCA 3 (Multimedia Appendix 1). Although this agency continued to print schedules rather than access them on the PDA, most health care personnel used the PDA in communication with managers and colleagues internally and with hospitals or general practitioners externally. Many workers remained ambivalent towards mobile technology. On one side, they saw the ability to gain information at the point of care and the telephone-and-text-message features as positive. One worker stated: “I think it is a major advance that we can now order medicines online.”

On the other hand, technical difficulties were a major source of frustration. Many care workers found it difficult to fill out records on the PDA, and some care workers were skeptical towards the utility of mobile technology. As one worker noted: “It cannot be of any good, except that by using the cell phone I can call the client if (s)he cannot hear the doorbell.”

#### Home Care Agency 3

This agency was actively engaged in designing an mHealth system as early as 2002 and, as a result, selected PDAs with offline connectivity. Computer Sciences Corporation (CSC) was the chosen provider. HCA 3 had registered services and working hours since the mid-1990s, and mobile technology was perceived by managers as a feasible way to facilitate and advance these activities. The key technology strategy was to improve “uniform level of service and contribute to documentation and transparency in the management of home care” [62].

Accordingly, care workers used mobile technology to access client information, look up schedules, and register working hours and services—as expected by managers that praised the new technology. However, sometimes care workers used the technology differently than planned by performing time registration at the end of their work day rather than “on the go”. Overall, the health care personnel perceived the monitoring and careful documentation of home care services as an unpopular system of control. The offline connectivity also proved controversial over time, and the personnel started to request more contemporary mobile devices with telephone features included. In response, HCA 3 established a pilot in 2007 based on government sponsorship. The agency decided, however, not to adopt online technology as an evaluation concluded that there were too many technical difficulties [63].

**Table 3:** Interpretive flexibility in mobile health across three cases.

	HCA 1	HCA 2	HCA 3
Nature of technology	Nokia Communicator with online connectivity and Zealand as provider.	PDAs with online connectivity and Ramböll as provider.	PDAs with offline connectivity and CSC as provider.
	Managers in support of mobile health.	Managers in support of mobile health.	Managers in support of mobile health.
	The reduction in meeting activity proved controversial for care workers, and many experienced difficulties filling out records.	Many care workers experienced technical difficulties, and some remained skeptical towards mobile technology.	Mixed attitude among care workers: many experienced increased control based on detailed time registration, and offline connectivity proved controversial.
Technology strategy	Mobile technology as coordination platform to share information and reduce meeting activities.	Mobile technology as communication medium to improve relationships and status of home care.	Mobile technology as management tool to improve documentation and transparency of service delivery.
Technology use	Use of mobile technology to support coordination by documenting and sharing information about activities. Joint morning meetings were abolished, as mobile technology afforded information exchanges.	Use of mobile technology to support communication with managers and colleagues internally and with hospitals or general practitioners externally.	Use of mobile technology to support management of resources by recording information about working hours and service delivery.

## Discussion

### Principal Findings

Our process analysis reveals how government sponsoring and collaboration between key stakeholders in the home care sector shaped the widespread adoption of mobile technology, whereas the case analyses demonstrate how mobile technology had interpretive flexibility with considerable variation in perceived nature of technology, technology strategy, and technology in use between the observed agencies. In the following section, we discuss these findings in detail.

First, strong collaboration between key stakeholders in the home care sector (eg, government bodies, vendors, consultants, interest organizations, and managers) helped initiate and energize the change process. The evidence suggests that mobile technology implementation in the Danish case was not only shaped within specific home care agencies, but also energized in a broader sociopolitical and interorganizational context [43]. Powerful stakeholders in the home care sector justified mobile technology usage; IT vendors and consultants inspired home care agencies, especially early on in the change process; and government accelerated the process by financing a pilot and sponsoring implementation across individual agencies. Indeed, the evidence suggests the widespread and relatively fast diffusion of mobile technology across Danish home care agencies was not only facilitated by, but to a large extent dependent on, these broader initiatives.

Second, the sector-wide pilot (CareMobile) served as an important arena for legitimizing use of mobile technology in home care agencies. As the CareMobile project unfolded from 2002 to 2005, it created a generic technology strategy and practical guidelines for using mobile technology to modernize home care. CareMobile involved interplay between key stakeholders across the home care sector that inspired design of possible technology in use scenarios and exchange of key lessons from mHealth. The pilot provided a sound business case for mHealth implementation and served as a significant inspiration for home care agencies. Moreover, CareMobile functioned as an important justification of subsequent government funding. In this sense, CareMobile represents an interesting example of how large-scale pilots can effectively influence widespread HIT implementation.

Third, government funding represented a double-edged sword. As evident in the process analysis, government sponsorship in 2006 facilitated swift diffusion of mobile technology within the sector and helped individual home care agencies engage in acquisition and implementation activities. The funding proved very successful and supported overall government strategies in the area [58]. Thus, this study corroborates findings from other studies [43] suggesting that public financing is an effective tool in supporting and diffusing new information technologies. However, the hasty diffusion accelerated by government sponsorship did not progress as a straightforward and unproblematic process. In the wake of the widespread diffusion, it became clear that mobile technology implementation was hampered by technical difficulties and associated with a system of organizational control. It is also likely that government funding for some agencies was the key driver behind the project (see Table 2) rather than an organizational need to use mobile technology. As mentioned in our interview with the representative from LGDK: “When you throw bags of money in front of the managers in home care, of course they attempt to grasp them.” Government funding was, in effect, an offer home care agencies could hardly refuse.

Fourth, mobile technology had interpretive flexibility and created considerable variation in how stakeholders perceived the nature of the technology, technology strategy, and technology use across home care agencies. Although the three home care agencies we observed all implemented mHealth systems, their approaches took quite different forms as the technology was transformed to fit the local context of each home care agency. They chose different vendors, the technology was perceived differently, they drew on different technology strategies, and they adopted different technology in use arrangements**.** For instance, whereas HCA 1 focused on mobile technology as a coordination platform, HCA 2 implemented mobile technology as a communication medium, and HCA 3 introduced mobile technology as a management tool. In this sense, we see how home care organizations are interpretive systems [64] and how mobile technology has considerable interpretative flexibility [36,37].

Fifth, while mobile technology in some instances was easily integrated with existing work practices, the integration was challenging in other instances. Mobile technology benefitted the health care personal in several ways. In line with other studies [5,10,11,16,26], our analyses demonstrate how health care personnel appreciated the ability to access client information at the point of care. At the same time, our study reveals mixed attitudes towards mobile technology among care workers. These different perceptions made mobile technology implementation more difficult than expected. For example, in HCA 1, the canceling of the joint morning meeting proved controversial as it conflicted with professional values, and in HCA 3, detailed time registrations were interpreted by care workers as a system of control. Accordingly, while the hopes of the transformative potential of mHealth are high, our research supports a more balanced view recognizing the challenges and difficulties in implementing mHealth systems [13-19].

### Conclusions

mHealth implementation does not appear to be a straightforward process with a clear beginning and end. Instead, our analyses demonstrate how mobile technology implementation was an interactive and muddled process that, like other aspects of contemporary health care organization, happened “in many places at once” [65]. Accordingly, we suggest that HIT implementation (such as mHealth) is most readily studied by combining organization-level analysis with features of the wider sociopolitical and interorganizational environment [42,43]. By conducting the overall process analysis, we were able to understand the political dynamics of mobile technology implementation. By moving to the organizational aspect in specific case studies, other aspects of the implementation process were given and demonstrated the interpretive flexibility of mHealth.

Our study provides valuable insights to decision makers and health care organizations as they engage in mHealth. The key lessons from the Danish case include the following: (1) participation and collaboration between a variety of stakeholder proved useful in supporting the implementation process, (2) government-sponsored programs can serve as double-edged swords, (3) managers need to be aware of the interpretive flexibility of mobile technology, and (4) mobile technology may in some areas collide with professional values and norms while in other areas being easily integrated in day-to-day working practices.

Although the mHealth initiative in Danish home care is an attractive subject for analysis as it represents a large-scale innovation project and includes comprehensive empirical data, it still represents only one example of mHealth implementation. As a result, some caution is required in generalizing our findings. Yet, there is a steadily increasing practice and research interest in how mHealth can be applied to improve health care delivery. While literature on mHealth often has relied on variance models such as the Technology Acceptance Model [66], we hope our study may encourage other scholars to include process and longitudinal investigations to more fully understand and draw lessons from mobile health.

## Multimedia Appendix 1

Supplementary tables.

## Abbreviations

CSC: Computer Sciences Corporation
HCA: home care agency
HIT: health care information technology
LGDK: Local Government Denmark
OECD: Organisation for Economic Co-operation and Development
PDA: personal digital assistant
