# Supplementary material for: Interpretive Flexibility in Mobile Health: Lessons From a Government-Sponsored Home Care Program
Source: J Med Internet Res. 2013 Oct 30;15(10):e236. doi: 10.2196/jmir.2816 (PMC3841343; doi:10.2196/jmir.2816)
Supplement: Supplementary file 1 [file jmir_v15i10e236_app1.pdf]

Multimedia Appendix 1: Supplementary tables.

Table A.1 – Mobile health technology adoption							
	**2004 (or before)**	**2005**	**2006**	**2007**	**2008***	**Non-adopters**	**Total**
Number of municipalities	8	10	22	36	13	9	98
%	8	10	22	37	13	9	100

Source: Home care mangers in all 98 municipalities were (in 2007) asked to specify when they adopted mobile technology among care workers.
*Estimate for 2008. Home care managers were (in 2007) asked if they expected care workers to adopt mobile technology during 2008. 13 managers answered yes to this question.

Table A.2 - Selected cases			
	**HCA 1**	**HCA 2**	**HCA 3**
Municipality Population (2008)	35.445	59.040	503.699
Geographical location	Jutland	Funen	Zealand
Number of care workers (2007)	270	400	3.300
Clients recieving home care (2007)	1.600	2.602	20.018
IT-provider	Zealand Care	Ramböll	CSC
Mobile device	Smartphone (Communicator)	PDA	PDA
Mobile devices in use (2008)	270	400	2.650
Implementation	2005	2005	2002-2007

Table A.3 - Care worker's use of mobile health technology. Percentage							
To what degree do you use mobile technology for…		To a high degree	To some degree	To a small degree	Not at all	SUM	N
Lookup work plan	HCA 1	92	3	0	5	100	36
	HCA 2	44	46	3	7	100	59
	HCA 3	76	18	4	2	100	84
Calling and SMS (telephone)	HCA 1	81	19	0	0	100	36
	HCA 2	71	12	12	5	100	58
	HCA 3	2	3	4	91	100	66
	HCA 1	20	49	23	8	100	35
	HCA 2	13	35	26	26	100	54

Access information at the point-of-care	HCA 3	35	40	19	6	100	80
Time registration	HCA 1	92	8	0	0	100	36
	HCA 2	5	15	13	67	100	39
	HCA 3	68	25	5	2	100	84
Writing journal notes	HCA 1	5	10	13	72	100	32
	HCA 2	5	27	20	48	100	56
	HCA 3	2	17	14	67	100	64

Note. Answer category " I don´t know" is omitted in the table.

Table A.4 - Care workers’ assessment of mobile health technology use. (%)

		Strongly agree	Agree	Disagree	Strongly disagree	SUM	N
Easy to use	HCA 1	24	64	6	6	100	33
	HCA 2	14	57	26	3	100	58
	HCA 3	25	51	16	8	100	81
Saves time	HCA 1	9	25	38	28	100	32
	HCA 2	9	28	34	29	100	53
	HCA 3	7	28	34	31	100	82
Better task [illegible]	HCA 1	3	40	34	23	100	35
	HCA 2	11	39	37	13	100	46
	HCA 3	11	35	32	22	100	78
Less stressful working day	HCA 1	6	6	49	39	100	33
	HCA 2	8	19	45	28	100	53
	HCA 3	4	10	58	28	100	73
Increases the control	HCA 1	27	46	27	0	100	33
	HCA 2	8	24	45	24	100	51
	HCA 3	30	31	34	5	100	80
Technical difficulties hinder use	HCA 1	20	62	15	3	100	34
	HCA 2	37	52	7	4	100	54
	HCA 3	19	53	24	4	100	74

Note. Answer category " I don´t know" is omitted in the table.
